# Evidence for shared neural information between muscle synergies and corticospinal efficacy

**DOI:** 10.1038/s41598-022-12225-1

**Published:** 2022-05-27

**Authors:** David R. Young, Caitlin L. Banks, Theresa E. McGuirk, Carolynn Patten

**Affiliations:** 1 Biomechanics, Rehabilitation, and Integrative Neuroscience (BRaIN) Lab, UC Davis School of Medicine, Sacramento, CA USA; 2 UC Davis Center for Neuroengineering and Medicine, University of California, Davis, Davis, CA USA; 3 VA Northern California Health Care System, Martinez, CA USA

**Keywords:** Motor control, Motor cortex, Neurological disorders, Neurophysiology

## Abstract

Stroke survivors often exhibit gait dysfunction which compromises self-efficacy and quality of life. Muscle Synergy Analysis (MSA), derived from electromyography (EMG), has been argued as a method to quantify the complexity of descending motor commands and serve as a direct correlate of neural function. However, controversy remains regarding this interpretation, specifically attribution of MSA as a neuromarker. Here we sought to determine the relationship between MSA and accepted neurophysiological parameters of motor efficacy in healthy controls, high (HFH), and low (LFH) functioning stroke survivors. Surface EMG was collected from twenty-four participants while walking at their self-selected speed. Concurrently, transcranial magnetic stimulation (TMS) was administered, during walking, to elicit motor evoked potentials (MEPs) in the plantarflexor muscles during the pre-swing phase of gait. MSA was able to differentiate control and LFH individuals. Conversely, motor neurophysiological parameters, including soleus MEP area, revealed that MEP latency differentiated control and HFH individuals. Significant correlations were revealed between MSA and motor neurophysiological parameters adding evidence to our understanding of MSA as a correlate of neural function and highlighting the utility of combining MSA with other relevant outcomes to aid interpretation of this analysis technique.

## Introduction

Stroke is the leading cause of physical disability in adults worldwide^1^. Gait dysfunction following stroke is widespread, persistent, and well described including decreased gait speed, increased paretic limb swing time, reduced paretic propulsion, and diminished peak ankle power^2–4^. Gait dysfunction compromises self-efficacy and negatively impacts autonomy, community participation, and quality of life^5,6^ motivating further investigation to better understand the interaction between supraspinal lesions, impaired descending commands, altered muscle activity, and resulting gait deficits^7–10^.

The computational resources required to directly control the muscle activation patterns required for gait are theoretically immense due to the numerous degrees of freedom in gait. Because the neurocomputational complexity involved exceeds feasibility for direct neural control of gait, research investigating physiological principles such as generalized motor programs and central pattern generators has contributed to determining how this complexity may be reduced to improve efficiency of motor control^11–13^. Muscle synergies, also called modules or factors, offer another theoretical framework for reducing computational complexity^10,14^. A synergy is comprised of a neural command (NC), that is a time-varying element which corresponds temporally to the movement patterns (here, the gait cycle), and a synergy vector (SV), a vector of the relative weighting coefficients for each recorded muscle^15^.

It has been argued that the product of a Muscle Synergy Analysis (MSA) characterizes the complexity of descending neural commands^16^. For example, it has been found that only five synergies are needed to account for 90% or more of the variance (VAF) in the accompanying muscle activity during gait^17,18^. When performed in populations with neuropathologies, MSA often identifies fewer synergies (NumSyn) required to achieve 90% or 95% VAF during gait, a finding which is frequently interpreted as reduced complexity of motor commands controlling gait^9,19^. For example, individuals with Parkinson’s Disease, Cerebral Palsy, and incomplete Spinal Cord Injury exhibit decreased NumSyn compared to healthy, age-matched peers^19–22^. This finding of fewer synergies in neuropathological conditions has been repeated in individuals following stroke, and these changes appear to be related to stroke severity^9,10,23–25^.

Despite recent popularization of MSA, its significance remains controversial^26^. Some researchers assert that MSA offers a direct correlate of neural function^16,27–29^. This notion has been supported by correlational neuroimaging analyses^30–33^. Alternatively, several investigations challenge the meaning and appropriate interpretation of MSA^34–36^. Experiments have modeled signals resembling synergies by accounting for only task constraints and muscular effort minimization without descending cortical commands^35^. Additionally, some modeled data that achieved a high VAF proved insufficient to achieve accurate movements, while others found that using biofeedback derived from kinetics, EMG, and muscle synergies led to similar movement outcomes﻿ ^34,36^. Collectively these studies highlight the practical utility of using a small set of synergies for motor coordinatio﻿n^37^. Adding to the controversy, a large number of methodological choices made during MSA impact the overall results and interpretation^15,38^. To date, no study has directly compared measures of corticospinal efficacy with MSA, which could provide insight and evidence regarding the significance of MSA, specifically the amount of shared neural information between these putative neuromarkers.

The presence, latency, and size of motor-evoked potentials (MEPs) generated by transcranial magnetic stimulation are broadly interpreted as measures of corticospinal efficacy^39^. MEP presence is related to cortical integrity; MEP latency serves as a marker of direct, monosynaptic versus less efficient indirect, oligosynaptic corticomotor responses; MEP size is related to cortical excitability^40,41^. Because MEP characteristics provide a direct marker of neurophysiological function, it is of interest to determine how MEP characteristics and MSA outcomes are related. Here we compared MEP characteristics and MSA outcomes between three groups of individuals to determine whether the characteristics of MSA, an indirect measure of nervous system function, are associated with biomechanical and neurophysiological function. Results of this investigation contribute to clarifying the scope within which MSA should be interpreted.

## Methods

### Subjects

Sixteen individuals with stroke (58.13 ± 7.95 years, F = 2) and eight age-matched control (65.56 ± 10.18 years, F = 5) participants were recruited to participate in this investigation (Table 1). Three groups were identified: High-Functioning Hemiparetic Individuals (HFH)—producing an average A2 value > 1.0 W/kg, Low-Functioning Hemiparetic individuals (LFH)—producing an average A2 of < 1.0 W/kg, and age-matched healthy participants (CON). Stroke survivors were included if their stroke was chronic (i.e., > 6 months), they exhibited motor impairment (i.e., hemiparesis), and they were able to walk independently at least 10 m with or without assistive devices. Exclusion criteria included bilateral or cerebellar lesions, other neurological, musculoskeletal, or cardiovascular dysfunction that limited walking ability, or severe cognitive deficits. Additional exclusion criteria included contraindications for transcranial magnetic stimulation (TMS), including implanted metal above the chest, seizure disorders, or pregnancy^42^. All procedures were approved by the University of Florida Health Science Center Institutional Review Board (IRB-01). Written informed consent was obtained from all participants prior to enrollment and all experimental procedures were conducted in accordance with the Declaration of Helsinki^43^. Testing was administered at the Brain Rehabilitation Research Center located at the Malcom Randall VA Medical Center (Gainesville, FL, USA).

**Table 1:** Demographic information.

	N	Age	Sex	Height (m)*	Weight (kg)	Chronicity (mo)	Paretic Side	Location	Mechanism	SSWS (m/s) *	FMA-LE*	A2 W/kg*
CON	8	58.13 ± 7.95	F = 5, M = 3	1.67 ± 0.08	76.15 ± 15.77	NA	NA	NA	NA	1.04 ± 0.14	NA	1.84 ± 0.15
HFH	8	66.63 ± 9.78	F = 1, M = 7	1.78 ± 0.06	85.29 ± 14.45	64.87 ± 38.54	R 5 L 3	Cortical 3 Subcortical 4 Mixed 1	Hemorrhagic 1 Ischemic 7	0.89 ± 0.09	33.62 ± 0.74	1.14 ± 0.09
LFH	8	64.5 ± 11.14	F = 1, M = 7	1.73 ± 0.07	81.18 ± 11.77	58.13 ± 55.35	R 3 L 5	Cortical 2 Subcortical 3 Mixed 3	Hemorrhagic 1 Ischemic 7	0.43 ± 0.11	24.25 ± 5.63	0.29 ± 0.11

Data include self-selected walking speed (SSWS), the lower extremity subscore of the Fugl-Meyer (FMA-LE) and peak ankle plantarflexor power (A2).

*Indicates significant differences between groups per an alpha value of p < 0.05.

### Procedures

Preamplified EMG electrodes (Motion Lab Systems, MA-420, Baton Rouge, LA, USA) were placed on 8 lower-limb muscles of each leg using SENIAM guidelines (Table 2)

**Table 2:** Bilateral EMG placement locations.

**Bilateral EMG locations**
Medial gastrocnemius (MG)
Soleus (SO)
Tibialis anterior (TA)
Vastus medialis (VM)
Rectus femoris (RF)
Biceps femoris (BF)
Gluteus medius (GMe)
Gluteus maximus (GMx)

^44^ alongside 14 mm reflective markers for motion capture placed to configure a modified Helen Hayes marker set^45^. Next, self-selected walking speed (SSWS) and baseline walking characteristics were identified while participants walked on an instrumented treadmill (Bertec, Columbus, OH, USA) wearing a fall arrest harness (Therastride, St. Louis, MO, USA; Robertson Harness Inc, Ft. Collins, CO, USA) for safety.

Next, participants were instrumented for TMS targeting the plantarflexor muscles using a custom batwing-shaped coil (90 mm, Magstim, Whitland, UK) oriented to induce current in a posterior-to-anterior direction and a Magstim 200^2^ device. After localization, the coil was stabilized using a custom-built helmet to maintain placement and counterbalance the weight of the coil and cable^46^. TMS was delivered at 120% of the active motor threshold (aMT) observed in quiet standing. Electrical stimulation (Estim) (Digitimer DS-7A/DS-7AH, Welwyn Garden City, UK) was delivered at the posterior tibial nerve to evoke motor responses and Hofmann reflexes (H-reflex) in the soleus (SO). Following the generation of H-reflex recruitment curves, Estim was delivered at the intensity level that produced an H-reflex at 50% of H-max.

Following instrumentation, participants walked at their self-selected walking speed (SSWS). Subject-specific gait events, detected using a combination of the vertical ground reaction forces and marker data acquired during baseline walking, were used to parameterize for TMS and Estim during the pre-swing phase (PSw) of gait approximately every third gait cycle. Experimental control was provided via custom-written Signal scripts (Version 6.0, Cambridge Electronic Design, Cambridge, UK). Here we report responses acquired using TMS delivered during the PSw of gait because this phase corresponds to  peak plantarflexor activity.

During walking, kinematic data were recorded by 12 near-infrared cameras (Vicon MX, Vicon Motion Systems Ltd., Oxford, UK; 200 Hz) and stored for offline analysis. Ground reaction forces, moments, and center of pressure were collected using a dual-plate instrumented treadmill (Bertec, Columbus, OH, USA). All analog signals were sampled at a rate of 2000 Hz.

### Data processing

Target leg ankle power was derived from kinematic and kinetic data and calculated using inverse dynamics (Visual3D Version 6, C-Motion, Germantown, MD). Ankle power in the sagittal plane was identified as the product of ankle joint moment and angular velocity^47^. The second peak of ankle power, A2, was calculated for each subject. Muscle Synergy Analysis (MSA) was performed using data derived from a minimum of ten but up to twenty gait cycles. Gait cycles including or immediately following either TMS or Estim events were disregarded for the MSA analysis. Raw EMG data were gain-corrected, converted to millivolts, demeaned, and filtered using a fourth-order zero-lag Butterworth bandpass filter (10–450 Hz). To perform MSA, EMG data were rectified and smoothed using a low-pass, fourth-order zero phase-lag Butterworth filter with a cutoff frequency of 7 Hz divided by the participant’s average stride time. Data were also time interpolated using force plate-derived gait events to obtain 101 samples per cycle^15^.

### Muscle synergy extraction

We performed MSA using non-negative matrix factorization (NNMF) to identify the underlying patterns of muscle activation following the methods described in Banks et al.^48^. EMG normalization entailed dividing each element of the EMG vector by the standard deviation of the entire trial (i.e., UnitOver)^15^. Each synergy derived from MSA produces a time-varying neural command (NC) and a set of weighting coefficients called synergy vectors (SVs): ${EMG}_{j}={SV}_{1j}{NC}_{1}+{SV}_{2j}{NC}_{2}+\dots +{SV}_{nj}{NC}_{n}+error$

In the above equation, EMG_j_ represents a matrix of the normalized EMG signals for *j* muscles. The scalar SVs and vector NCs can be linearly combined to reconstruct the original EMG. With *j* muscles and *n* < *j* synergies, the reconstructed EMG matrix will be lower-dimensional than the original input matrix^49^.

SVs were normalized to their maximum value (i.e., SV Max)^15^. We identified the variance accounted for (VAF) by each synergy, as well as NumSyn required to reach a VAF of 90%. Given NumSyn’s small range, for correlational analyses VAF explained by the first synergy was used in lieu of NumSyn. All analysis was performed using custom MATLAB scripts (The MathWorks r2020a, Natick, MA, USA).

### Neurophysiological parameter extraction

Motor evoked potential (MEP) latency was identified as the time from stimulation until a signal threshold (baseline activity ± 1SD) was crossed. MEP area was calculated as all activity exceeding the signal threshold. A similar process was employed to identify H-reflex latency. The difference between SO MEP and H-reflex latencies (latency difference) was calculated for each subject (MEP_latency_ − H-reflex_latency_). Because we analyzed MEPs resulting from stimulations delivered during gait, substantial background muscle activity was observed. As a result, all neurophysiological data were reviewed and verified with user-interaction.

### Statistical analysis

Demographic and biomechanical outcomes, and NumSyn, were compared between groups using univariate analyses of variance (ANOVAs). A Group by Synergy ANOVA compared VAF within the first five synergies, as five synergies were sufficient to explain over 99% of the VAF in our sample. Group by Muscle ANOVAs were performed to identify differences in SVs between groups in specific synergies related to plantarflexion, dorsiflexion, and hip extension. Statistical Parametric Mapping (SPM) compared NCs between groups (spm1d v0.4.7 for MATLAB, Institute of Neurology, London, UK)^50^. Two-way ANOVAs for NCs related to plantarflexion, dorsiflexion, and hip extension were performed. In the event of a significant finding, the Bonferroni corrected significance level for SPM was p < 0.017. For all other analyses, statistical significance was identified as p < 0.05.

MEP latency and area of the MG, SO, and TA were compared between groups using One-Way ANOVAs. Finally, Spearman correlation analyses were computed between the VAF explained by the first synergy and behavioral outcomes as well as MEP latencies and areas. Main effect sizes were derived as partial eta squared (﻿η_p_^2^). VAF explained by the first synergy was used instead of NumSyn due to NumSyn’s small range and non-normal distribution. In the case of a significant main effect, pairwise comparisons were made. Hedge’s G effect sizes were used for significant pairwise comparisons. Statistical comparisons were made using SPSS 26 (IBM, Armonk, NY, USA) and SPM.

## Results

### Subjects

Data from 24 participants were included. Results from a univariate ANOVA confirmed that there was no difference in age between groups (p = 0.11 η_p_^2^ = 0.19). Demographic information can be found in Table 1﻿. Data are reported as mean ± standard deviation.

### Clinical and biomechanical outcomes

**Figure 1:**
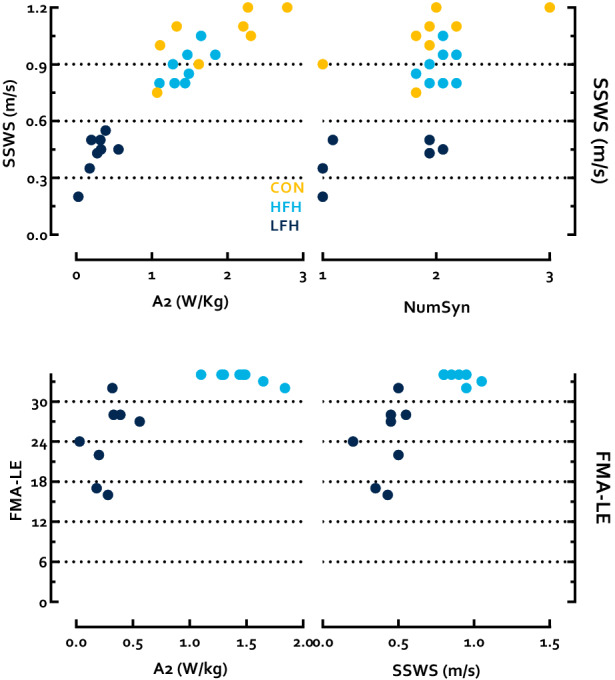
Top left Individual plots of A2 and SSWS, (top right) NumSyn and SSWS, (bottom left) A2 and the FMA-LE, and (bottom right) SSWS and the FMA-LE for CON (gold), HFH (light blue), and LFH (dark blue).

Differences between groups in self-selected walking speed were found (p < 0.0001 η_p_^2^ = 0.84). Pairwise comparisons identified that the CON group walked faster than both HFH and LFH individuals (p = 0.02 HG = 1.19; p < 0.0001 HG = 4.57). HFH individuals also walked faster than LFH (p < 0.0001 HG = 4.54). Similarly, A2 differed between groups (p < 0.0001 η_p_^2^ = 0.75), with CON and HFH outperforming LFH, while CON and HFH did not differ (p < 0.0001 HG = 3.32; p < 0.0001 HG = 5.89; p = 0.07, respectively). HFH individuals also exhibited higher scores than LFH on the lower extremity subsection of the Fugl-Meyer Assessment (FMA-LE) (p = 0.002 HG = 2.34) (Fig. 1). These data are presented in Table 2.

### Muscle synergy analysis

**Figure 2:**
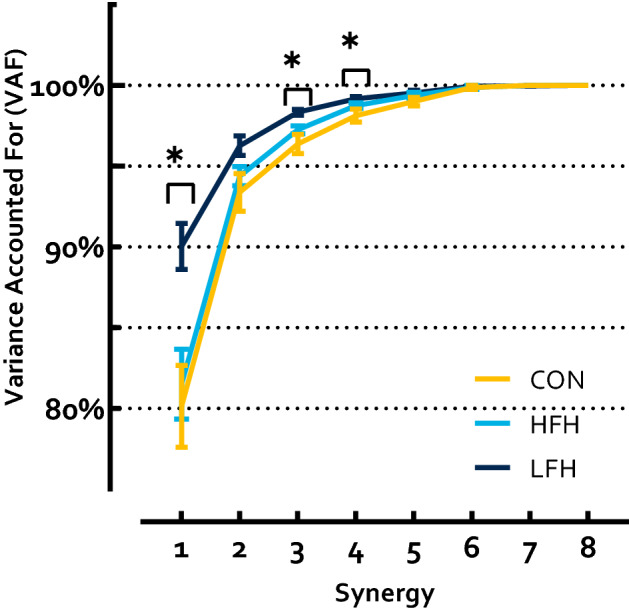
VAF accounted for by each synergy. LFH individuals exhibited greater VAF in the 1st synergy than CON and HFH, and in the 3rd, and 4th synergies compared to CON. HFH and CON did not differ. Asterisks denote a p < 0.05.

To satisfy a 90% VAF threshold (i.e., NumSyn), the CON group required 2.0 ± 0.53 synergies, HFH individuals required 2.0 ± 0.0, and LFH individuals required 1.38 ± 0.52 synergies (p = 0.01 η_p_^2^ = 0.75). CON and HFH did not differ, while both CON and HFH exhibited a greater NumSyn than LFH (p = 1; p = 0.008 HG = 1.19; p = 0.008 HG = 1.71, respectively). Results of a Group by Synergy ANOVA revealed a significant main effect of Synergy on VAF (p < 0.0001 η_p_^2^ = 0.86), a significant Group effect (p = 0.003 η_p_^2^ = 0.43) and a Synergy by Group Interaction effect (p = 0.008 η_p_^2^ = 0.33) (Fig. 2). Follow-up comparisons were performed to identify which synergies differed between groups. In the first synergy, VAF was lower in CON and HFH than LFH, while CON and HFH did not differ (p = 0.0026, η_p_^2^ = 0.39; CON and LFH p = 0.009 HG = 1.70; HFH and LFH p = 0.02 HG = 1.65; CON and HFH p = 1). No between group differences were identified in the VAF attributed to the second synergy (p = 0.065 η_p_^2^ = 0.23). VAF within the third synergy also differed between groups (p = 0.007 η_p_^2^ = 0.38). CON exhibited lower VAF than LFH, while HFH differed from neither CON nor LFH (p = 0.005 HG = 1.59; p = 0.38; p = 0.18, respectively). The same was true in the fourth synergy (p = 0.029 η_p_^2^ = 0.29; p = 0.03 HG = 1.26; p = 0.31; p = 0.75, respectively). No differences were identified between groups within the fifth synergy (p = 0.21 η_p_^2^ = 0.14).

**Figure 3:**
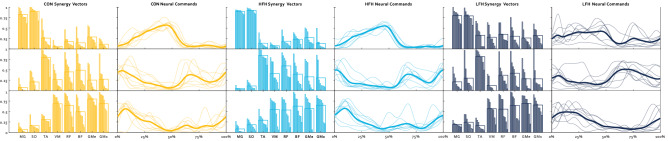
Synergies for CON (gold), HFH (light blue), and LFH (dark blue). The top row corresponds to the Plantarflexion Synergy, the middle row to the Dorsiflexion Synergy, and the bottom row to the Support Synergy. The 1st, 3rd, and 5th columns correspond to Synergy Vectors and the 2nd, 4th, and 6th columns to Neural Commands. Individual participants are represented by thin bars and lines, while thicker bars and lines represent group averages.

In order to compare the composition of muscle synergies between groups, we extracted NCs and SVs from the three dominant synergies found in our sample: Plantarflexion, Dorsiflexion, and Hip Extension. The Plantarflexion Synergy weighted plantarflexors highest and was most active during the stance-to-swing transition. The Dorsiflexion Synergy weighted the TA highest and was most active during swing. The Hip Extension Synergy weighted the quadriceps, hamstrings, and gluteal muscles highest and was active primarily at the beginning and end of the gait cycle ( Fig. 3). SVs were compared between groups for each Synergy. Results of a Group by Muscle ANOVA sought to identify differences in SVs between groups, and found that the Plantarflexion Synergy SVs were significantly different between groups (p = 0.035 η_p_^2^ = 0.35), however there was no significant Muscle by Group interaction (p = 0.13 η_p_^2^ = 0.43). Pairwise comparisons revealed that SVs were not different between Control and HFH individuals, while LFH differed from both CON and HFH (p = 1; p = 0.018; p = 0.04, respectively). There was no effect of group on SVs in either the Dorsiflexion Synergy or the Hip Extension Synergy (p = 0.74 η_p_^2^ = 0.03; p = 0.58 η_p_^2^ = 0.03).

SPM identified a significant difference between groups in the NC of the Plantarflexion Synergy (p = 0.03). The groups differed from 0 to 3% and from 90 to 100% of the time-normalized gait cycle (p = 0.03; p = 0.001). However, follow-up t-tests with a corrected alpha value of p = 0.017 failed to confirm differences between CON and HFH, CON and LFH, or HFH and LFH (p > 0.05; p = 0.04; p > 0.05, respectively). No differences were observed between groups in the Dorsiflexion Synergy or Hip Extension Synergy NCs (p’s > 0.05).

### Correlation between MSA and other outcomes

Spearman correlation identified that VAF by the first synergy was significantly associated with A2 (r = − 0.45 p = 0.03) as well as SSWS (r = − 0.57 p = 0.003). Within stroke survivors, VAF by the first synergy was related to the FMA-LE (r = − 0.58 p = 0.02). VAF by the first synergy was also related to MEP latencies in each tested muscle (MG r = 0.42 p = 0.04; SO r = 0.45 p = 0.03; TA r = 0.46 p = 0.02), as well as MEP area in the MG (r = − 0.55 p = 0.006) and SO (r = − 0.67 p < 0.001), but not the TA (r = − 0.31 p = 0.14). VAF by the first synergy was not correlated to the latency difference (r = 0.3 p = 0.15) (See Fig. 7).

### Neurophysiology

**Figure 4:**
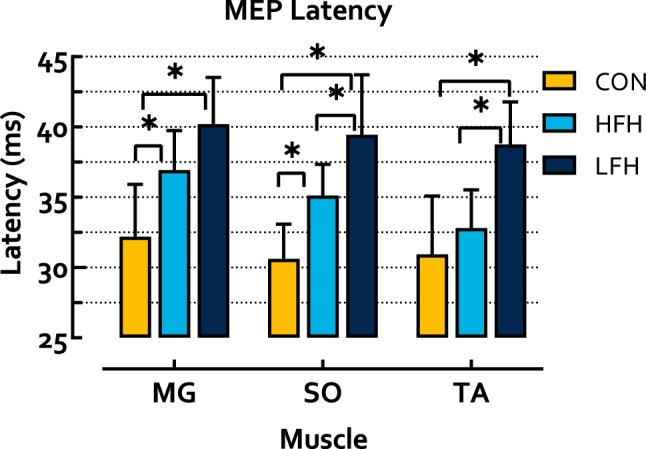
MEP latency in the shank muscles during walking. CON exhibited significantly shorter latencies than both HFH and LFH individuals. HFH exhibited shorter latencies than LFH in the SO and TA. Asterisks denote a p < 0.05.

**Figure 5:**
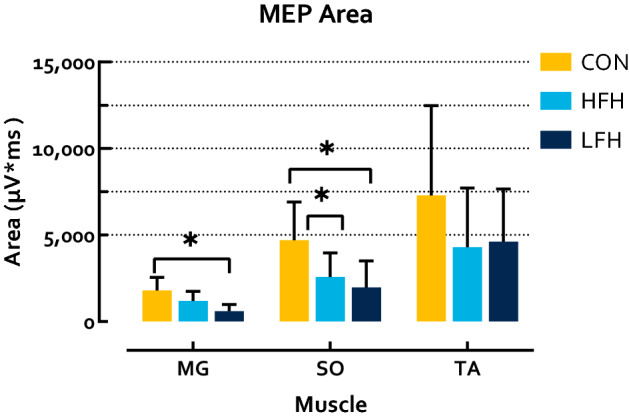
MEP Area in response to TMS during walking. CON exhibited greater MEP area than LFH individuals in the MG, as well as LFH and HFH individuals in the SO. No differences were observed in the TA. Asterisks denote a p < 0.05.

**Figure 6:**
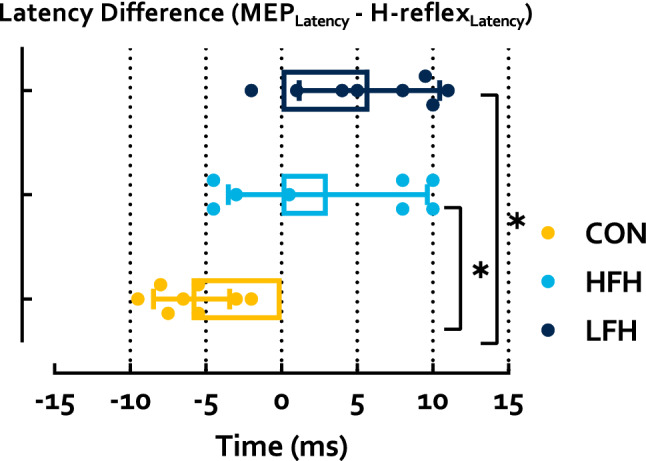
Latency difference derived using SO MEP latency and H-reflex latency. This relationship was significantly more negative in CON than LFH or HFH, while LFH and HFH did not differ. Asterisks represent a p < 0.05.

MEP latency differed significantly between groups for each tested muscle (MG p < 0.001 η_p_^2^ = 0.53; SO p < 0.001 η_p_^2^ = 0.60; TA p < 0.001 η_p_^2^ = 0.53). Follow-up analyses identified that CON exhibited shorter latencies than HFH in the MG (p = 0.009 HG = 1.45) and SO (p = 0.009 HG = 1.92), but not the TA (p = 0.28), while CON exhibited shorter latencies than LFH in each muscle (MG p < 0.001 HG = 2.26; SO p < 0.001 HG = 2.53; TA p < 0.001 HG = 2.15). HFH exhibited shorter latencies than LFH in the SO (p = 0.001 HG = 1.27) and TA (p = 0.002 HG = 2.07), but not the MG (p = 0.06) (Fig. 4). MEP area differed significantly between groups in the MG and SO, but not TA (MG p = 0.007 η_p_^2^ = 0.41; SO p = 0.03 η_p_^2^ = 0.31; TA p = 0.30 η_p_^2^ = 0.12). Follow-up analyses identified that CON did not differ from HFH (MG p = 0.16; SO p = 0.10; TA p = 0.43) while CON exhibited greater area than LFH in the MG and SO, but not TA (MG p = 0.006 HG = 1.93; SO p = 0.04 HG = 1.36; TA p = 0.83). HFH and LFH did not differ (MG p = 0.33; SO p = 1; TA p = 1) (Fig. 5). The difference between SO MEP latency and H-reflex latency also differed between groups (p < 0.001 η_p_^2^ = 0.55). CON exhibited a latency difference of − 5.34 ± 2.51 ms, indicating that conduction time from TMS to MEP onset was less than H-reflex latency. Conversely, both stroke subgroups exhibited MEP latencies greater than H-reflex latency (HFH 3.06 ± 6.58 ms; LFH 5.81 ± 4.64 ms). CON exhibited shorter latency difference than HFH and LFH, while HFH and LFH were not significantly different (p = 0.001 HG = 1.81; p < 0.001 HG = 2.98; p = 0.27) (Fig. 6).

## Discussion

**Figure 7:**
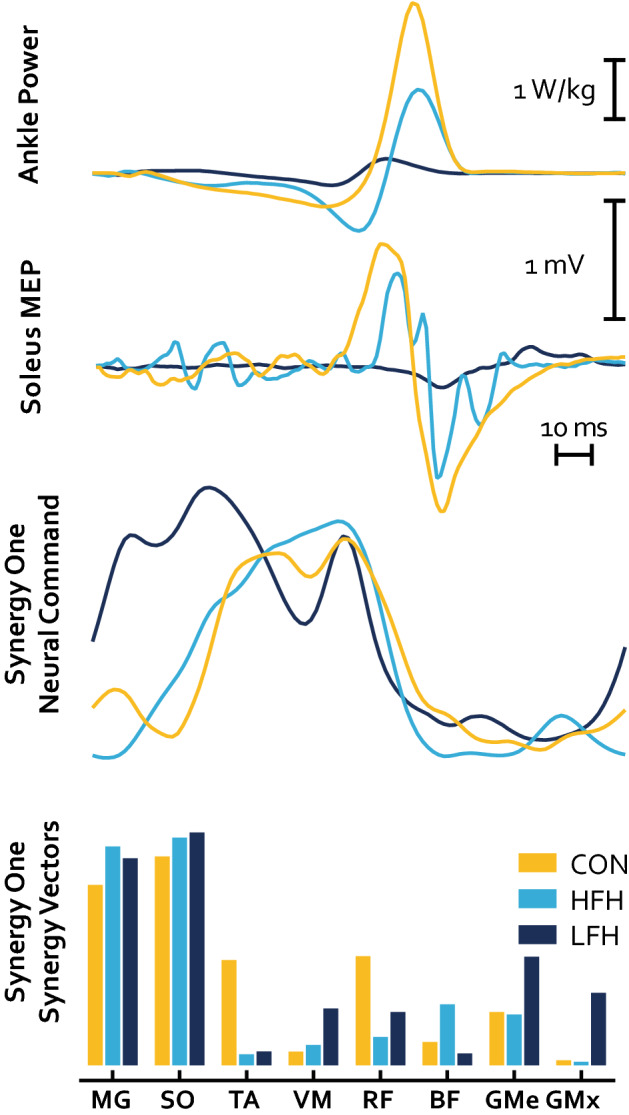
Example behavior of representative subjects for CON (gold), HFH (light blue), and LFH (dark blue). The 1st row represents ankle power, the 2nd row represents an MEP derived from TMS during the pre-swing phase of walking, the 3rd row is a representative Plantarflexion Neural Command over the full gait cycle, and the 4th row is a representative Plantarflexion Synergy Vector.

In this investigation, we compared CON, HFH and LFH individuals following stroke. We used single-pulse TMS and Estim to probe corticospinal efficacy between stroke survivors and control participants during locomotion. Results of this investigation add to the body of literature comparing MSA outcomes in healthy individuals and stroke survivors, as well as the literature comparing MSA outcomes to clinical and biomechanical factors influencing gait. Furthermore, for the first time, this investigation performed both MSA and MEP analyses from data acquired concurrently in the same sample, allowing for inferences regarding their relationship and the presence of shared neural information between these two metrics (Fig. 7).

Previously, researchers have successfully differentiated healthy individuals and stroke survivors by NumSyn^9,10,23,24^. We identified a greater NumSyn in the CON and HFH groups than LFH. However, NumSyn was unable to distinguish between CON and HFH. The lack of differences may be partially explained by the fact that HFH individuals in this study were relatively high-functioning (e.g., SSWS 0.89 ± 0.09 m/s, Lower-Extremity Fugl-Meyer (FMA-LE) 33.62 ± 0.74 out of 34). Furthermore, HFH did not exhibit significantly lower A2 than CON, suggesting that their gait biomechanics are minimally impaired. Nevertheless, our results suggest that NumSyn may be somewhat less sensitive than differences identified between the CON and HFH in SSWS and neurophysiological parameters.

NumSyn is often employed in MSA, however this measure is subject to investigator decision-making; for example, the choice between a 90% or 95% threshold can impact results. Here, while using a threshold of 90% did identify that LFH required fewer synergies than CON, had we used a threshold of 95%, no differences would have been identified between any of the three groups in the current sample (p = 0.12 η_p_^2^ = 0.18). These results coupled with the fact that accounting for a high percentage of variance does not inherently lead to successful movement patterns suggests that researchers must be cautious both when selecting a critical threshold and interpreting the results of MSA^36,51^. A possible alternative may be to perform a comparison of the VAF by each synergy. In the current experiment, a Group by Synergy ANOVA identified significant main effects of group as well as a significant group by synergy interaction effect, with specific differences within several synergies. Comparing groups based on the VAF added by each synergy also distinguished between CON and LFH individuals, as well as HFH and LFH individuals without relying on investigator-made decisions regarding a critical threshold. A similar approach was used by Bekius et al., who found that children with cerebral palsy exhibit greater VAF in the first synergy in their more-affected side^52^. Alternatively, Ballarini et al. described an algorithm for choosing the optimal NumSyn, which may also serve to decrease the impact of investigator choice on the results^53^. Recent work has challenged commonly used assumptions of MSA analysis, suggesting the presence of physiologically relevant data in the residual activity commonly attributed to noise^51^. Furthermore, extracting synergies beyond commonly utilized VAF thresholds (e.g., 90–95%) has been found to influence the reconstructed movement patterns^51^. In the current study, we compare groups using both NumSyn as well as VAF by each synergy. Further investigation into methods to distinguish noise from task-relevant signal at, or above, a pre-defined VAF threshold is warranted.

Differences in SVs were observed between groups in the Plantarflexion Synergy. CON and HFH individuals did not differ, while muscle weightings were altered in LFH individuals. No group differences were observable in the Dorsiflexion or Hip Extension synergies. The ability to detect differences within SVs between groups is a source of some controversy. For example, children with cerebral palsy did not exhibit altered SVs compared to typically developing children^54^. Similarly, SVs were not different following sub-acute stroke, or in Parkinson’s Disease^20,28^. Conversely, some researchers have identified differences between SVs in healthy subjects and individuals with incomplete spinal cord injury^17,22,55^. Regardless of whether differences in SV composition can be statistically identified, questions remain regarding whether such differences provide novel or physiologically important information. Roh et al. found increased coactivation among the three heads of the deltoid muscles in stroke survivors during upper extremity tasks, leading to altered SVs (i.e., increased weighting of the deltoids)^56^. Greater coactivation within synergies has been found in other experiments as well^19,57,58^. As other EMG analysis techniques can be employed to measure co-contraction indices without the need to consider muscle synergies, or the need to identify the investigator-dependent decisions required to conduct and interpret the results of an MSA, these may be more attractive options until the investigator-dependent decisions become standardized^38,47^.

Like Synergy Vectors, Neural Command comparisons have led to a wide array of results. Several investigations have found no differences in NCs between groups despite obvious physiological differences including differences in NumSyn^22,28,55^. Gizzi et al. argued that the lack of differences between groups in their investigation indicate NCs may be preserved in stroke due to the maintained functionality of the spinal cord^28^. Conversely, investigations that have found differences in NCs between groups have applied several methods to do so. NC duration has been compared between groups by quantifying the amount of time the NC signal surpasses a given threshold of signal maximum^19,54^, or alternatively, the time-index of the NC’s signal maximum^20,59^. We utilized Statistical Parametric Mapping to identify whether group differences occurred at any point in the NC time-series. Visual inspection suggested that LFH individuals may not deactivate the Plantarflexor Synergy as readily as CON or HFH individuals. However, after statistical corrections for multiple comparisons, pairwise comparisons did not reach the threshold of significance. Furthermore, no differences were found in the Dorsiflexion or Hip Extension synergies. The relatively small number of individuals in each group may contribute to low statistical power. Regardless, this result highlights the need to minimize investigator decision-making by standardizing how NCs are compared, as it may strongly influence whether the groups can be differentiated.

This investigation also sought to examine measures of corticospinal efficacy in isolation. TMS was applied during walking using a custom built helmet to secure the coil in place^46^. As a result, we were able to probe corticospinal efficacy during the relevant task of locomotion. The CON group exhibited the shortest latency in MG and SO MEPs, producing a negatively signed latency difference, indicative of MEPs occurring prior to the H-reflex. Latency information aids inference regarding whether the pathway from the motor cortex to the muscle fibers is monosynaptic, or requires multiple synapses, thus serving as a biomarker for corticospinal efficacy^40,41^. SO MEP area was also greater in CON than individuals with stroke, with no distinction between HFH and LFH. No differences were detected between groups in TA MEP area. This may be the case for several reasons. First, the current investigation analyzed data derived from TMS delivered during the pre-swing phase (PSw) of walking, which is characterized primarily by plantarflexor activity with little input from dorsiflexors. Second, because plantarflexor function is intimately linked with gait function^2,60^ the TMS coil was localized to evoke plantarflexor MEPs. While MEPs were often observed in the TA, TMS pulses did not directly target the TA and, as a result, TA MEPs exhibited considerable within-and-between subject variability.

The current analysis did not reveal differences between CON and HFH in NumSyn, VAF by each synergy, SVs, or NCs. Nevertheless, we found a significant correlation between NumSyn and both SSWS and A2 in the whole sample as well as the FMA-LE in stroke survivors. VAF by the first synergy was also related to MEP latency in all tested muscles and MEP area in the MG and SO, demonstrating that while MSA was unable to differentiate between CON and HFH, it was related to corticospinal efficacy, walking speed, and paretic A2 in this sample. The high level of function in the HFH group, as well as the sample size of eight individuals per group may partially explain the lack of MSA differences between CON and HFH.

Controversy already exists regarding the appropriate interpretation of MSA^26,61^. In 2013, de Rugy et al. argued that VAF is a relatively insensitive measure, finding that while four synergies were able to achieve 90% VAF in healthy participants performing an upper limb task, outcomes of modeled data derived from those four synergies led to poor performance of their model. This finding led the group to question the utility of measuring VAF because high VAF can occur in the presence of poor motor performance^36^. Furthermore, Kutch & Valero-Cuevas (2012) and De Groote et al. (2014), were both able to model signals that resembled muscle synergies without accounting for descending commands from the cortex. Kutch & Valero-Cuevas modeled muscle activity based solely on anatomical characteristics and task constraints^34^ while De Groote et al. modeled muscle activation based on task-constraints including a goal to minimize overall muscle activity^35^.﻿ Both authors’ efforts created signals which resembled muscle synergies.﻿ As a result, these authors argued that it was incorrect to attribute signals known as muscle synergies solely to descending neural commands from the cortex. Instead, these signals emerge from the interaction of task-constraints, central pattern generators, and sensory feedback without the need for descending signals^35^.

Serving as a counterpoint to the above arguments, recently, multiple authors have reported a relationship between cortico-muscular coupling and MSA outcomes in both humans and non-human primates. Liu et al. found decreased strength of cortico-muscular coupling after stroke was related to NumSyn and SV differences compared to healthy individuals^33^. Overduin et al. identified that features of muscle synergies, including the dimensionality, timing (NCs), and weightings (SVs), could be observed both at the cortical and muscular level in primates^31^.

In conclusion, we found that TMS administered during walking yielded MEPs that differentiated between controls and stroke survivors. MEP latency and area were different, as was the latency difference. This investigation highlights the feasibility of generating MEPs during walking and provides an avenue for future investigation comparing MEPs in task-relevant situations. We also found that while no MSA outcome differentiated controls from HFH individuals, LFH individuals differed significantly from CON in NumSyn and the Plantarflexion Synergy SV measures, but not NC measures. Nevertheless, we found statistical associations between MSA outcomes and neurophysiological parameters of corticospinal efficacy suggesting there may be some shared neural information between these metrics of neuromotor function. MSA calculation methods, including manipulation of outcomes for post-processing comparisons are highly dependent on investigator decisions. While it is possible to identify differences between groups in each measure, especially within NumSyn, the utility of MSA to provide novel, relevant, unbiased information regarding neuromotor dysfunction has yet to be conclusively demonstrated. Therefore, interpretation and application of MSA should be performed in conjunction with biomechanical or neurophysiological parameters known to reflect motor dysfunction in order to improve the interpretability of results.

## Data Availability

The datasets generated during and/or analyzed during the current study are available from the corresponding author on reasonable request.
